# Cysteine-Rich Secretory Proteins (CRISP) are Key Players in Mammalian Fertilization and Fertility

**DOI:** 10.3389/fcell.2021.800351

**Published:** 2021-12-14

**Authors:** Soledad N. Gonzalez, Valeria Sulzyk, Mariana Weigel Muñoz, Patricia S. Cuasnicu

**Affiliations:** Instituto de Biología y Medicina Experimental (IByME-CONICET), Ciudad Autónoma de Buenos Aires, Buenos Aires, Argentina

**Keywords:** sperm, egg, gamete, fertilization, fertility, CRISP

## Abstract

Mammalian fertilization is a complex process involving a series of successive sperm-egg interaction steps mediated by different molecules and mechanisms. Studies carried out during the past 30 years, using a group of proteins named CRISP (Cysteine-RIch Secretory Proteins), have significantly contributed to elucidating the molecular mechanisms underlying mammalian gamete interaction. The CRISP family is composed of four members (i.e., CRISP1-4) in mammals, mainly expressed in the male tract, present in spermatozoa and exhibiting Ca^2+^ channel regulatory abilities. Biochemical, molecular and genetic approaches show that each CRISP protein participates in more than one stage of gamete interaction (i.e., cumulus penetration, sperm-ZP binding, ZP penetration, gamete fusion) by either ligand-receptor interactions or the regulation of several capacitation-associated events (i.e., protein tyrosine phosphorylation, acrosome reaction, hyperactivation, etc.) likely through their ability to regulate different sperm ion channels. Moreover, deletion of different numbers and combination of *Crisp* genes leading to the generation of single, double, triple and quadruple knockout mice showed that CRISP proteins are essential for male fertility and are involved not only in gamete interaction but also in previous and subsequent steps such as sperm transport within the female tract and early embryo development. Collectively, these observations reveal that CRISP have evolved to perform redundant as well as specialized functions and are organized in functional modules within the family that work through independent pathways and contribute distinctly to fertility success. Redundancy and compensation mechanisms within protein families are particularly important for spermatozoa which are transcriptionally and translationally inactive cells carrying numerous protein families, emphasizing the importance of generating multiple knockout models to unmask the true functional relevance of family proteins. Considering the high sequence and functional homology between rodent and human CRISP proteins, these observations will contribute to a better understanding and diagnosis of human infertility as well as the development of new contraceptive options.

## Introduction

In mammals, sperm that leave the testes are not capable of recognizing and fertilizing the egg. In order to acquire fertilizing competence, sperm must undergo several physiological changes during their transit through the male and female reproductive tracts, known as sperm maturation (Robaire and Hinton, 2015) and sperm capacitation (Chang, 1951; Austin, 1952), respectively. Whereas sperm maturation occurs during epididymal transit and confers sperm the ability to move progressively and to fertilize the egg, sperm capacitation takes place while sperm are ascending through the female tract towards the oviduct and allows sperm to undergo both the acrosome reaction, an exocytotic event that occurs in the head, and to develop a vigorous flagellar movement termed hyperactivation. Both the acrosome reaction and hyperactivation are essential for the gamete interaction process that occurs in the oviductal ampulla and which involves several coordinated and successive stages (i.e., cumulus penetration, zona pellucida (ZP) sperm-ZP binding, ZP penetration, gamete fusion) (Florman and Fissore, 2015).

For more than 30 years, our laboratory has been dedicated to elucidate the molecular mechanisms involved in the mammalian fertilization process using as a model the evolutionarily conserved Cysteine-RIch Secretory Protein (CRISP) family, a group of highly homologous proteins enriched mainly in the mammalian reproductive tract and the venom of reptiles (Mochca-Morales et al., 1990; Yamazaki and Morita, 2004; Gibbs and O’Bryan, 2007). The CRISP family, together with the Antigen-5 and the Pathogenesis Related-1 proteins, forms the CAP superfamily of proteins found in a wide range of organisms including humans. The tertiary structure of CAP proteins shows a remarkable conservation despite significant phylogenetic distance between organisms, suggesting that these proteins may be involved in common and essential biological processes (Gibbs et al., 2008). CRISP proteins (Mw 20–30 kDa) are characterized by the presence of sixteen conserved cysteines, ten of which are located in the C-terminal region or cysteine-rich domain (CRD), which is connected by a hinge region to the plant pathogenesis-related 1 (PR-1) domain located in the N-terminus (Guo et al., 2005; Gibbs et al., 2008). Whereas the N-terminal domain was proposed to be involved in cell-cell adhesion and fusion (Maeda et al., 1998; Ellerman et al., 2006) as well as in amyloid-type aggregation and/or oligomerization (Sheng et al., 2019; Sheng et al., 2021), the C-terminal domain showed the ability to regulate various ion channels (i.e., Cyclic Nucleotide Gated (CNGs), Ryanodine (RyR), Transient Receptor Potential ion channel Member 8 (TRPM8), Cation channel of Sperm (CatSper), etc.) (Yamazaki et al., 2002; Gibbs et al., 2006, 2011; Ernesto et al., 2015). In mammals, four CRISP proteins have been identified, mainly expressed in the male reproductive tract. The following sections describe their functional roles during the fertilization process as well as their relevance for animal fertility.

## Evidence on the Relevance of CRISP Proteins for Fertilization and Fertility Through the Use of Non-genetic Approaches

### CRISP1

CRISP1, the first member of the family, was identified in the rat epididymis and originally named DE (Cameo and Blaquier, 1976). It is an androgen-dependent glycoprotein that associates with the sperm plasma membrane during epididymal transit (Kohane et al., 1980a, 1980b; Garberi et al., 1982; Eberspaecher et al., 1995) with two different affinities (Cohen et al., 2000a). Whereas a major loosely bound population is released during capacitation, acting as a decapacitating factor and preventing a premature capacitation in the male tract (Cohen et al., 2000a; Roberts et al., 2003), a minor, strongly bound population, remains in sperm after capacitation, migrates from the dorsal region of the acrosome to the equatorial segment concomitant with the acrosome reaction and participates in gamete interaction (Rochwerger et al., 1992; Cohen et al., 2000b). Biochemical and molecular approaches revealed that CRISP1 plays a role in both sperm binding to the ZP and gamete fusion through its binding to complementary sites located in the ZP and the egg plasma membrane (oolemma), respectively (Rochwerger et al., 1992; Cohen et al., 2000b; Busso et al., 2007a). Considering that gamete fusion involves a first stage of sperm binding to the oolemma followed by a subsequent step of fusion between the sperm and egg plasma membranes, it is interesting to note that CRISP1 was found to participate in a step subsequent to sperm binding to the oolemma and leading to gamete fusion through a small region of only 12 amino acid residues that resides in an evolutionary conserved region of the whole CRISP family called Signature 2 (Ellerman et al., 2006). It is important to mention that the human homologue (hCRISP1) (Haendler et al., 1993; Hayashi et al., 1996; Krätzschmar et al., 1996), like its rodent counterpart, is also expressed in the epididymis, binds to human sperm with two different affinities and participates in both sperm binding to the ZP and gamete fusion through complementary sites localized in the human egg (Cohen et al., 2001; Maldera et al., 2014). In this regard, whereas evidence showed that ZP3 acts as a ZP binding site for CRISP1 (Maldera et al., 2014), the identity of binding sites for CRISP proteins involved in gamete fusion remains unknown. Although originally described in males, CRISP1 is also expressed along the female reproductive tract (Reddy et al., 2008) including the cumulus cells that surround the egg where it also plays a role in gamete interaction and, more specifically, in cumulus penetration, through its ability to modulate sperm motility and orientation (Ernesto et al., 2015).

Interestingly, electrophysiological studies revealed that CRISP1 also exhibits the ability to regulate TRPM8, a thermo sensitive Ca^2+^ channel located in both the sperm head and tail plasma membranes, and proposed to regulate the progesterone- and ZP-induced acrosome reaction (Martínez-López et al., 2010; Ernesto et al., 2015) as well as CatSper, the main sperm Ca^2+^ channel located in the principal piece of the tail and essential for sperm hyperactivation and male fertility (Ren et al., 2001; Sun et al., 2017). In this regard, although several inhibitors of CatSper have been described (Rennhack et al., 2018), to our knowledge, CRISP1 is the only physiological blocker of CatSper described so far.

The first evidence of the potential relevance of CRISP1 not only for fertilization but also for fertility was observed when male and female rats were immunized with either native or recombinant CRISP1. This strategy produced high levels of antibodies and a significant decrease in fertility in both sexes without eliciting pathological effects (Cuasnicu et al., 1990; Perez Martinez et al., 1995; Ellerman et al., 1998, 2008; Muñoz et al., 2012). These results, later supported by plasmid based contraceptive vaccines encoding mouse CRISP1 (Luo et al., 2012, 2016), constitute a strong proof of concept that blocking epididymal protein CRISP1 by immunological or pharmacological means, may lead to an effective male contraceptive. Moreover, immunization of non-human primates with hCRISP1 also produced specific antibodies that enter the male reproductive tract, recognize the native protein on sperm and remain associated with the ejaculated cells without eliciting effects on sperm number, morphology and motility, excluding deleterious effects of the immune response on the testes and/or the epididymis (Ellerman et al., 2010). This, together with the inhibitory effect of anti-hCRISP1 antibodies on both human sperm-ZP interaction and gamete fusion (Cohen et al., 2001; Maldera et al., 2014), supports CRISP1 as a promising contraceptive target in men. Thus, CRISP1 seems to fulfill many of the criteria to be considered an attractive target for contraception as it is an epididymal protein localized in the surface of mammalian sperm being accessible to immunological or drug attack, it has key functional roles in fertilization (i.e gamete interaction and CatSper regulation), it is relevant for fertility, and it has a functional homologue in humans.

### CRISP2

CRISP2, initially identified as testicular protein-1 (TPX-1) (Kasahara et al., 1989), is a non-glycosylated protein expressed almost exclusively in the testis in an androgen independent manner (Haendler et al., 1997) and present in germ cells of numerous species (i.e., guinea pig (Hardy et al., 1988), rat (Maeda et al., 1998; O’Bryan et al., 1998), mouse (Kasahara et al., 1989; Mizuki et al., 1992), human (Kasahara et al., 1989), horse (Giese et al., 2002) and boar (Vadnais et al., 2008)). CRISP2 localizes in the surface of spermatogenic cells (Maeda et al., 1998) and within the sperm acrosome (O`Bryan et al., 2001; Nimlamool et al., 2013), neck and outer dense fibers of the tail (O'Bryan et al., 1998, 2001). Like CRISP1, CRISP2 also relocalizes to the equatorial segment after acrosome reaction (Cuasnicu et al., 2016). However, while CRISP1 migrates to the equatorial segment, CRISP2 is released from the acrosome and then associates to the surface of the equatorial segment (Busso et al., 2005; Busso et al., 2007a; Muñoz et al., 2012; Nimlamool et al., 2013). Interestingly, recent results showed that under native, non-reducing conditions, CRISP2 formed oligomers both in the tail and the head but with different molecular weights and different biochemical properties (Zhang et al., 2021). Although a slight expression of CRISP2 has been observed in the ovary (Reddy et al., 2008) the relevance of this observation is still unknown.

Structure and function studies revealed that while the N-terminal domain of CRISP2 exhibits cell to cell adhesion properties (Maeda et al., 1999), the C-terminal domain is able to regulate Ca^2+^ RyR channels (Gibbs et al., 2006). Considering that both RyR (Harper et al., 2004) and CRISP2 are located in the neck and that the Ca^2+^ released from intracellular stores at the neck is involved in sperm hyperactivation (Chang and Suarez, 2011), it is likely that CRISP2 modules sperm hyperactivation by regulating RyR controlling intracellular Ca^2+^ stores. As previously described for CRISP1, CRISP2 located in the equatorial segment of acrosome reacted sperm (Busso et al., 2005, 2007b; Muñoz et al., 2012; Nimlamool et al., 2013) also participates in gamete fusion through its interaction with egg plasma membrane complementary sites (Busso et al., 2005; Busso et al., 2007b). Interestingly, competition studies indicate that CRISP2 binds to the same egg complementary sites that CRISP1 (Busso et al., 2007b), suggesting that CRISP2 may cooperate with CRISP1 during gamete fusion. Whereas this cooperation might be due to a synergistic action of each individual protein, the possibility cannot be excluded that these two proteins could eventually form a complex (i.e., dimers and/or oligomers) to achieve that role, consistent with the reported oligomeric properties of CRISP family members (Zhang et al., 2021). Human CRISP2 (Kasahara et al., 1989) was also found to be present within the sperm acrosome and tail and to participate in gamete fusion through complementary sites in the human egg plasma membrane (Busso et al., 2005).

In contrast to the significant inhibition of fertility observed in animals injected with CRISP1 (Ellerman et al., 1998, 2010), immunization of male and female rats with recombinant CRISP2 raised specific antibodies in both sexes without affecting animal fertility (Muñoz et al., 2012), consistent with the internal localization of CRISP2. Interestingly however, evidence indicates that aberrant CRISP2 expression is associated with human fertility problems. Patients with azoospermia or oligoasthenoteratospermia (Du et al., 2006) or with asthenospermia (Jing et al., 2011; Heidary et al., 2019) syndromes exhibit lower expression of CRISP2 than fertile man (Gholami et al., 2020). Moreover, a correlation was found between CRISP2 expression and low sperm progressive motility, abnormal sperm morphology and infertility, suggesting that the lower expression of CRISP2 in these patients could be due to a post-transcriptional regulation process mediated by miR27 b (Zhou et al., 2015). In agreement with the observations in humans, recent studies revealed a positive correlation between CRISP2 expression levels and boar fertility and that sperm CRISP2 has the potential to serve as a fertility biomarker (Gao et al., 2021).

### CRISP3

CRISP3, originally described in mice salivary glands (Haendler et al., 1993) and in human neutrophil granules (Kjeldsen et al., 1996), is expressed in an androgen-dependent manner (Schwidetzky et al., 1995; Haendler et al., 1997) and, unlike the other members of the CRISP family, shows a wider expression distribution including exocrine glands such as pancreas and prostate (Kratzschmar et al., 1996), organs with immunological roles such as the thymus and spleen and, at lower levels, the epididymis, seminal vesicle, ovary and uterus (Kratzschmar et al., 1996; Schambony et al., 1998; Evans et al., 2015). In humans, CRISP3 was described in several tissues and differences in its expression are associated with different pathologies such as prostate cancer (Kosari et al., 2002; Bjartell et al., 2007; Noh et al., 2016), breast cancer (Tang et al., 2020), Sjögren’s syndrome (Laine et al., 2007), varicocele (Belardin et al., 2019), prostatitis and endometriosis (Grande et al., 2017), among others. Two forms of human CRISP3 (i.e. glycosylated and non-glycosylated) were described along the male reproductive tract (Ubdy et al., 2005) and found to bind to human sperm with different affinities (Da Ros et al., 2015). While the glycosylated form is weakly bound and released during capacitation, the non-glycosylated form is tightly bound and remains on the spermatozoa even after the acrosome reaction (Da Ros et al., 2015), similarly to the two populations described for CRISP1. CRISP3 was also found to be present in horse seminal plasma and to prevent the interaction between polymorphonuclear cells and spermatozoa in the uterus (Doty et al., 2011), supporting a possible role in sperm protection during their transit through the female reproductive tract. However, little information exists about the relevance of CRISP3 for fertilization (Da Ros et al., 2015) and fertility (Hamann et al., 2007). Although no specific role of CRISP3 in ion channel regulation has been reported, the low levels of this protein in lacrimal and salivary gland secretion of patients with Sjögren’s syndrome (Tapinos et al., 2002; Laine et al., 2007) together with the altered ion concentrations reported in these glands (Enger et al., 2014) known to be relevant for their functionality (Konttinen et al., 2006), support the idea that CRISP3 may influence the ion concentration of these glands, likely through an ion channel regulatory ability.

### CRISP4

CRISP4 is an androgen-dependent protein almost exclusively synthesized in the epididymis and not expressed in the female reproductive tract (Jalkanen et al., 2005; Reddy et al., 2008; Turunen et al., 2012). Like CRISP1, epididymal CRISP4 associates with sperm during maturation (Jalkanen et al., 2005; Nolan et al., 2006). However, differently from CRISP1, CRISP4 is strongly bound to sperm and remains on the cells even after the acrosome reaction, lacking a loosely bound population (Weigel Muñoz et al., 2019). Patch-clamp of murine testicular sperm revealed that the CRD domain of CRISP4 has the ability to inhibit TRPM8 without affecting capacitation-associated parameters (i.e., sperm tyrosine phosphorylation or progesterone-induced acrosome reaction) (Gibbs et al., 2011). These observations together with the lack of a loosely bound population support a role for CRISP4 in gamete interaction rather than as a decapacitating factor as previously proposed for CRISP1. The first evidence on the involvement of CRISP4 in sperm-egg interaction emerged when two different groups developed the CRISP4 null mice and will be described in the following section (Gibbs et al., 2011; Turunen et al., 2012). Interestingly, mice fed a high-fat diet present a decline in sperm motility and fertilization in part from the disruption of epididymal CRISP4 expression and secretion (Borges et al., 2017). Rodent CRISP4 was found to exhibit high homology with epididymal hCRISP1, even higher than that between the rodent and human CRISP1 protein, suggesting that CRISP4 represents the rodent counterpart of hCRISP1 (Jalkanen et al., 2005; Nolan et al., 2006; Arevalo et al., 2020). However, subsequent observations showing the involvement of CRISP4 in gamete interaction led to propose that hCRISP1 is the equivalent to the combination of rodent CRISP1 and CRISP4 (Gibbs et al., 2011; Maldera et al., 2014).

In summary, the reported observations indicate that CRISP proteins exhibit a high sequence, structural and functional homology being involved in different stages of the fertilization process. To better analyze their functional roles as well as their relevance for fertility, our laboratory and others have generated several knockout models for CRISP family members (i.e., single, double and multiple knockouts) which exhibit different phenotypes described in detail in the following section.

## Evidence on the Relevance of CRISP Proteins for Fertilization and Fertility Through the Use of Knockout Models

### Single Knockout Models

#### Crisp1^−/−^

In addition to the immunization studies showing the need of CRISP1 for fertility and, as another approach to study the relevance of this protein for fertility, our laboratory developed the CRISP1 knockout mice which represented the first knockout animal for a CRISP family member (Da Ros et al., 2008). Surprisingly, animal fertility was not affected in either male or female mice (Da Ros et al., 2008; Ernesto et al., 2015), and the same results were later obtained in knockout male mice for CRISP1 generated in different genetic backgrounds (Hu et al., 2018; Weigel Muñoz et al., 2018), revealing that blocking the protein in an adult animal by immunization differs from deleting the gene, an approach that allows functional compensation of the lacking molecule during animal development. Nevertheless, in spite of their normal fertility, knockout males showed sperm with clear defects to interact with the ZP and to fuse with the egg (Da Ros et al., 2008), consistent with the previously proposed roles of CRISP1 in fertilization (Rochwerger et al., 1992; Busso et al., 2007a). Comparison of phenotypes in CRISP1 knockout mice of different genetic background revealed new roles for CRISP1 in hyperactivation development, sperm motility, progesterone-induced acrosome reaction and cAMP/PKA signaling pathway (Weigel Muñoz et al., 2018). Interestingly, whereas defects in gamete interaction, hyperactivation and cAMP/PKA signaling seem to withstand the genetic contexts, progesterone-induced acrosome reaction, motility and tyrosine phosphorylation defects were clearly dependent on the genetic background of the mutant animals (Weigel Muñoz et al., 2018), indicating that the phenotype observed in CRISP1 null males is not entirely controlled by the mutation at *Crisp1* locus but it is modulated by the genetic context. Of note, in spite of the Ca^2+^ channel regulatory activity described for CRISP1 (Ernesto et al., 2015), intracellular Ca^2+^ levels seem to be normal in *Crisp1* ^*−/−*^ sperm (Weigel Muñoz et al., 2018). Given the reported ability of CRISP1 to affect both CatSper currents and intracellular Ca^2+^ levels (Ernesto et al., 2015), it is possible that the lack of CRISP1 produces a Ca^2+^ deregulation which is compensated by other CRISP family members and, thus, not reflected in the total concentration of the cation within the cell (Weigel Muñoz et al., 2018). In addition to our observations in males, the availability of CRISP1 knockout females confirmed that CRISP1 is also expressed along the female reproductive tract (i.e., uterus, oviduct and ovary), including the cumulus cells that surround the egg (Ernesto et al., 2015) where CRISP1 was proposed to orient sperm by regulating hyperactivation through its ability to inhibit CatSper (Ernesto et al., 2015).

#### *Crisp2* ^*−/−*^

The proposed role of CRISP2 in gamete interaction (Busso et al., 2005; 2007b) together with the reported association between fertility defects and aberrant expression of CRISP2 in humans (Du et al., 2006; Jing et al., 2011; Zhou et al., 2015) led our laboratory to generate CRISP2 knockout mice. However, as observed for CRISP1, males lacking CRISP2 were fertile under controlled laboratory conditions (Brukman et al., 2016) supporting the existence of a functional compensation of the lacking molecule by other members of the family. Nevertheless, fertility evaluation under more demanding conditions such as the use of *Crisp2^−/−^* males subjected to unilateral vasectomy to reduce sperm number in the ejaculate (Judd et al., 1997), showed a significant decrease in fertility rates compared to controls (Brukman et al., 2016). Consistent with this, a slight subfertility was observed in CRISP2 knockout males generated in a different genetic background (Lim et al., 2019), indicating that CRISP2 is indeed necessary for optimal fertility.

Apart from the relevance of CRISP2 for fertility, the analysis of CRISP2 knockout animals allowed a better understanding of the role of this protein in sperm physiology. *In vivo* studies revealed that unilaterally vasectomized *Crisp2* ^*−/−*^ mice also showed lower levels of fertilized eggs in the ampulla, confirming that fertilization defects were responsible for the lower fertility rates observed in these mice (Brukman et al., 2016). In addition, whereas no differences in *in vivo* fertilization were observed when mutant males were mated with natural estrus females, significantly lower *in vivo* fertilization rates were observed for *Crisp2* ^*−/−*^ males mated with hormone-stimulated females that ovulate a higher number of eggs compared to estrus females, representing a more demanding condition for mutant sperm (Brukman et al., 2016). According to these results, it is clear that whereas control males could deal with different *in vivo* modifications, *Crisp2* ^*−/−*^ males could not. These observations may be extrapolated to humans where the subfertility of an individual can or cannot be detected depending on the fertility status of the partner.

In addition to the *in vivo* fertilization defects, CRISP2 knockout sperm exhibited defects to fertilize ZP-free eggs *in vitro* (Brukman et al., 2016), consistent with the reported role of CRISP2 in gamete fusion through egg plasma membrane complementary sites in both rodents and humans (Busso et al., 2005; 2007b). However, a more pronounced decrease in fertilization rates was observed when *in vitro* experiments were performed using eggs surrounded by the cumulus and/or the ZP, supporting a role for CRISP2 in penetration of the egg coats. In agreement with this, a lower number of *Crisp2* ^*−/−*^ sperm was observed within the cumulus mass during cumulus penetration assays, and hyperactivated motility was significantly lower in mutant sperm (Brukman et al., 2016). Subsequent studies supported that these motility defects could be due to a stiffness of the midpiece in CRISP2 mutant sperm that impairs hyperactivation development and thus, egg coat penetration (Lim et al., 2019; Curci et al., 2020). Different from *Crisp1* ^*−/−*^ animals, sperm lacking CRISP2 exhibit a clear dysregulation of Ca^2+^ homeostasis (Brukman et al., 2016) that could explain the molecular mechanisms underlying the capacitation-associated defects in the mutant cells. In this regard, recent results in CRISP2 knockout mice support the idea that ion channel regulation by CRISP proteins controls energy flows powering the axonema (Nandagiri et al., 2021). Moreover, CRISP1, CRISP2, and CRISP4 have been proposed to be required to optimize sperm flagellum waveform (Gaikwad et al., 2021).

Together, our *in vivo* and *in vitro* results reveal that CRISP2 knockout mice exhibit clear fertilization deficiencies likely linked to defects in hyperactivation development and intracellular Ca^2+^ regulation, supporting that fertilization defects may be underlying the fertility disorders observed in men with aberrant expression of CRISP2. Of note, the finding that reproductive defects in CRISP2 knockout mice are masked by conventional mating, becoming evident under more demanding conditions, highlights the relevance of using different experimental approaches to analyze male fertility.

#### *Crisp3* ^*−/−*^

In contrast to the rest of the CRISP family members, there is little information on the relevance of CRISP3 for fertilization and fertility. Although a recent work reports that *Crisp3* ^*−/−*^ males were fertile, no further details on the reproductive phenotype of these animals were provided (Volpert et al., 2020). In this regard, the phenotype exhibited by mice lacking both *Crisp1* and *Crisp3* (see below) does not support a critical role for CRISP3 in fertilization.

#### Crisp4^−/−^

*Crisp4* single knockout males were also found to be fertile even when generated using different strategies and genetic backgrounds (Gibbs et al., 2011; Turunen et al., 2012; Carvajal et al., 2018; Hu et al., 2018). As mentioned above, the availability of CRISP4 knockout mice allowed the study of the participation of CRISP4 in the fertilization process which had not been studied before. *Crisp4* ^*−/−*^ males exhibited normal fertilization rates in eggs recovered from the ampulla of superovulated females (Carvajal et al., 2018), indicating that *Crisp4* ^*−/−*^ sperm, differently from *Crisp2* ^*−/−*^ cells, are able to fertilize control eggs even in demanding conditions such as those produced by the presence of a high number of eggs in the ampulla. However, sperm lacking CRISP4 exhibited a severely affected ability to fertilize cumulus oocyte complexes as well as ZP-intact and ZP-free eggs under *in vitro* conditions (Carvajal et al., 2018). In this regard, whereas there are reports on the involvement of CRISP4 in sperm-ZP binding (Turunen et al., 2012), subsequent assays showing that CRISP4 mutant sperm were able to penetrate the cumulus cells and bind to the ZP together with the lack of accumulation of sperm in the perivitelline space support that fertilization defects in this colony may also reside at ZP penetration (Carvajal et al., 2018). Although sperm lacking CRISP4 did not exhibit defects in hyperactivation, the lower levels of progesterone induced-acrosome reaction observed in these cells compared to controls (Carvajal et al., 2018) could well explain their impaired ability to penetrate ZP-intact eggs as the acrosome reaction is essential for egg coat penetration (Yanagimachi, 1994). Interestingly, whereas a significant (Gibbs et al., 2011) or complete (Turunen et al., 2012; Carvajal et al., 2018) inhibition of progesterone-induced acrosome reaction was observed in all CRISP4 knockout models (Gibbs et al., 2011; Turunen et al., 2012; Carvajal et al., 2018), normal percentages of acrosome reacted sperm were observed in response to calcium ionophore, a non-physiological inductor of the acrosome reaction (Turunen et al., 2012), suggesting that CRISP4 might regulate the acrosome reaction by affecting calcium transport while not affecting the successive stages after calcium influx has occurred. Considering that the recombinant CRISP4 CRD domain has shown to be able to inhibit TRPM8 Ca^2+^ channel (Gibbs et al., 2011), it is possible that a modification in intracellular Ca^2+^ concentration would be responsible for the altered capacitated-sperm parameters observed in *Crisp4* ^*−/−*^ sperm. Acrosome reaction defects observed in all knockout models might also contribute to the impaired gamete fusion ability of CRISP4 knockout sperm as only acrosome-reacted sperm are able to fuse with the oolemma. Nevertheless, the possibility that CRISP4 mediates gamete fusion through a ligand-receptor interaction as previously observed for epididymal CRISP1 cannot be excluded.

**FIGURE 1:**
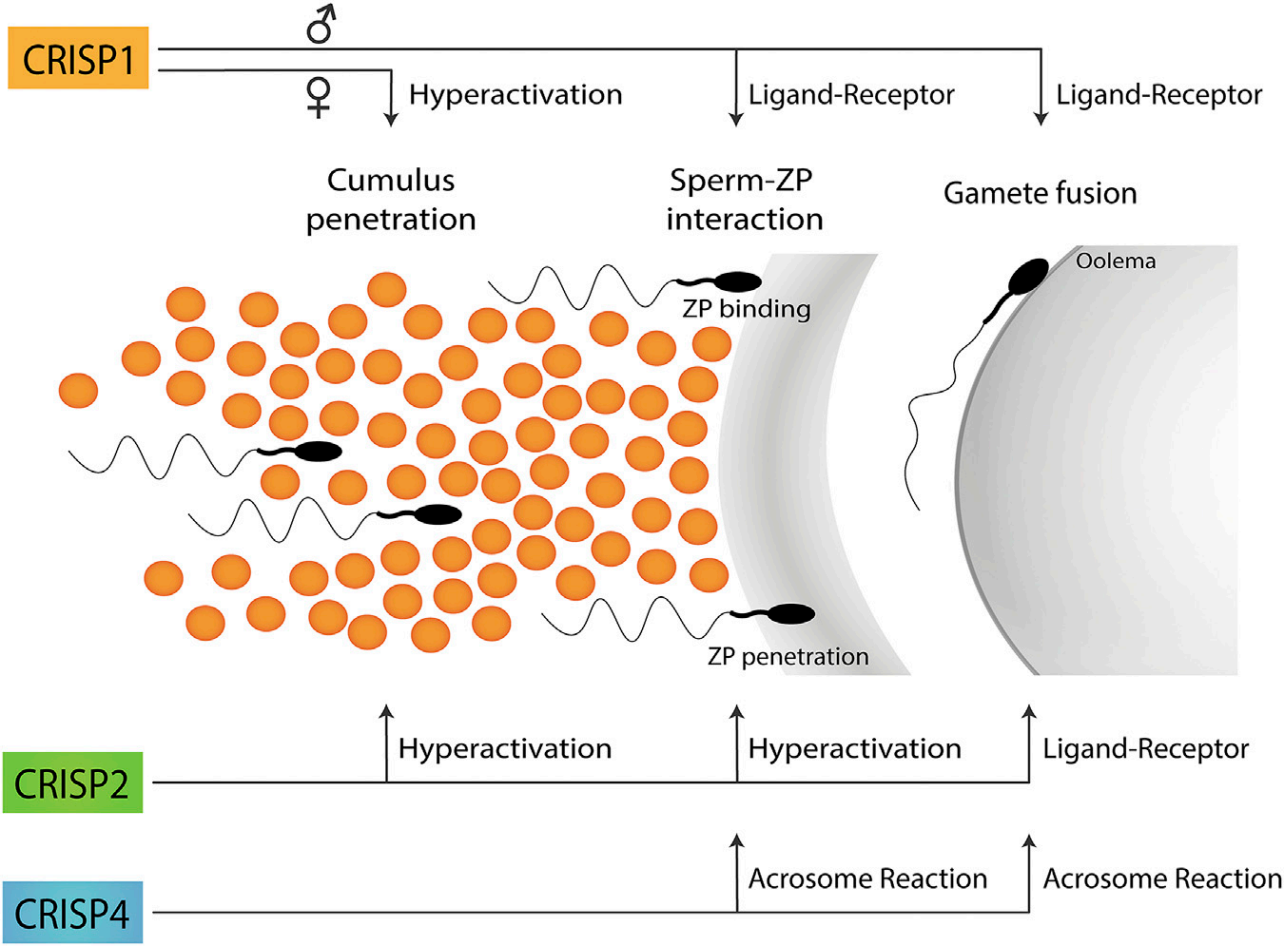
Participation of CRISP proteins in different steps of the fertilization process. Epididymal CRISP1 was reported to participate in both sperm-ZP binding and gamete fusion through ligand-receptor interactions whereas cumulus CRISP1 was proposed to play a role in cumulus penetration by orienting sperm through its ability to modulate sperm hyperactivation. Testicular CRISP2 was reported to be involved in both cumulus and ZP penetration through its ability to regulate sperm hyperactivation as well as in gamete fusion through ligand-receptor interactions. Finally, CRISP4 was reported to play a role in sperm-ZP interaction as well as in gamete fusion consistent with its ability to regulate the acrosome reaction. No information is still available on the functional roles of CRISP3 in gamete interaction. A schematic sperm representation is included.

**FIGURE 2:**
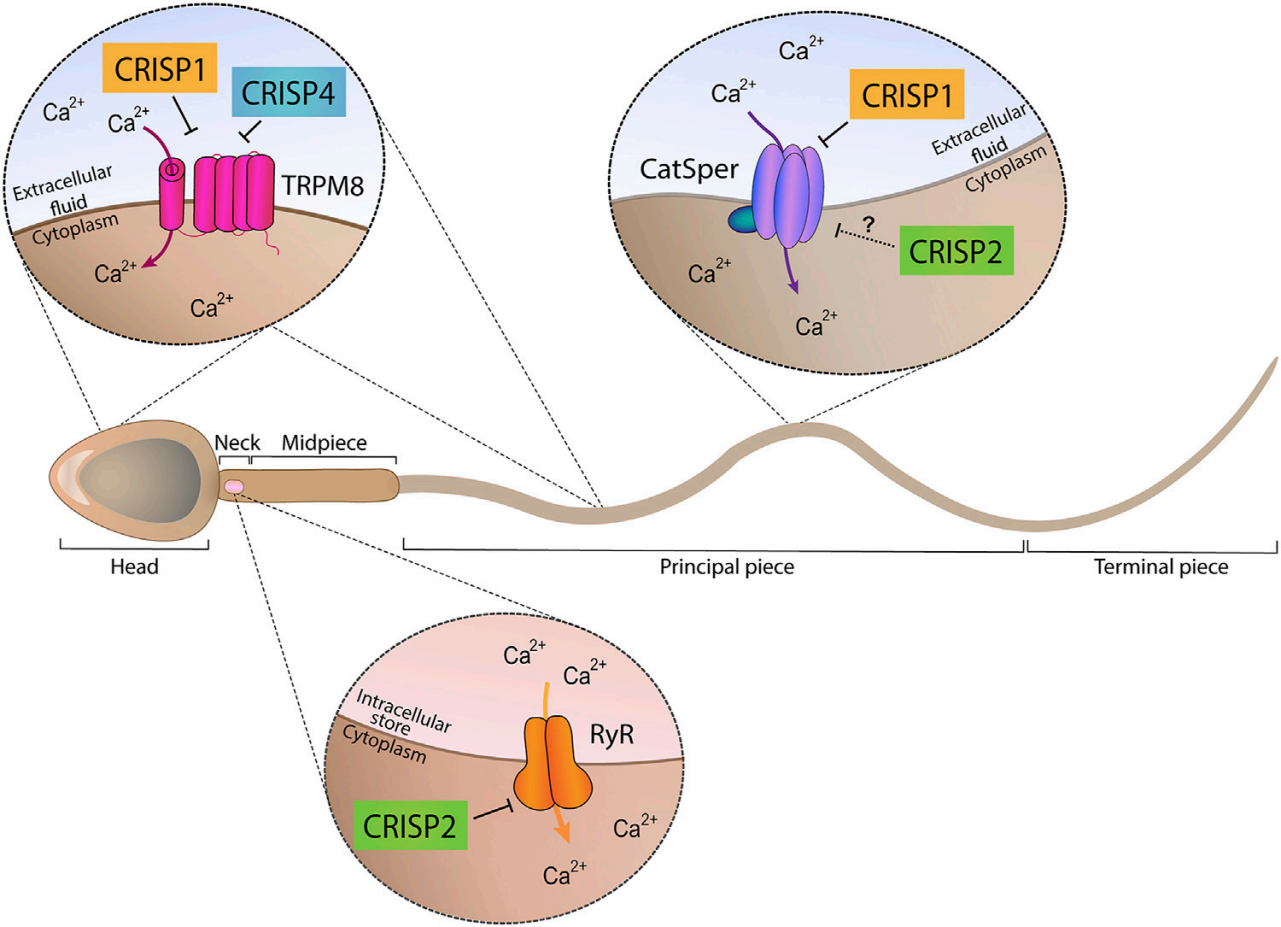
Ion channel regulatory abilities of CRISP proteins. Epididymal proteins CRISP1 and CRISP4 (located in the head and tail plasma membrane) were found to regulate TRPM8 channel present in both sperm head and tail plasma membranes and proposed to be involved in acrosome reaction regulation. In addition, epididymal CRISP1 was found to exhibit the ability to regulate CatSper, located in the principal piece of the tail and involved in hyperactivation development. Testicular CRISP2 (located within the sperm head, neck and tail) was found to regulate Ryanodine Receptors (RyR) known to be present in the neck and to control intracellular Ca^2+^ stores. A schematic sperm representation is included.

Taken together, the results obtained using single knockout models support the participation of each CRISP protein in more than one stage of fertilization and the involvement of more than one CRISP in each stage of the fertilization process either by ligand-receptor interactions or by regulating different functional events (i.e., acrosome reaction, hyperactivation, etc.) (Figure 1), likely through their ability to regulate different sperm Ca^2+^ channels (Figure 2).

However, in spite of the critical roles of CRISP proteins in different stages of fertilization, all single knockout animals were fertile, supporting the existence of functional overlapping or redundancy among CRISP family members. In this regard, the development of mutant mice has revolutionized the reproductive field showing that only a small number of the proteins previously known to play an essential role in the fertilization process were indeed essential for fertility in mice (Miyata et al., 2016). A possible explanation for these observations may be the existence of functional redundancy among protein members of the same family which could partially or totally compensate for each other’s loss, contributing to strengthening reproductive success. Such a mechanism becomes especially important in sperm which are transcriptionally and translationally silent cells. Based on this, animals simultaneously lacking more than one CRISP were generated and characterized. The following sections describe the different phenotypes observed for doubles, triples and quadruples knockout mice for *Crisp* family genes.

### Double Knockout Models

#### Crisp2^−/−^/Crisp4^−/−^

The fact that *Crisp2* and *Crisp4* are located in different chromosomes together with the availability of CRISP2 and CRISP4 single knockout colonies, led our laboratory to generate double *Crisp2* ^*−/−*^ */Crisp4* ^*−/−*^ mice by natural mating (Curci et al., 2020). Analysis of animal fertility revealed that, in spite of the participation of both proteins in key stages of the fertilization process, males lacking both proteins were fertile under normal laboratory conditions (Curci et al., 2020). Nevertheless, when *Crisp2* ^*−/−*^ */Crisp4* ^*−/−*^ males were mated under more demanding conditions such as using superovulated females, a clear decrease in *in vivo* fertilization rates was observed compared to controls and to either *Crisp2* or *Crisp4* single mutant males (Curci et al., 2020). Consistent with this and with the reported roles of CRISP2 and CRISP4 in different stages of the fertilization process, *Crisp2* ^*−/−*^ */Crisp4* ^*−/−*^ sperm showed severe *in vitro* fertilization defects likely due to the combination of the capacitation-associated defects observed in sperm from each single knockout model (i.e. impaired tyrosine phosphorylation, progesterone-induced acrosome reaction and hyperactivation development). Although the reasons for the normal fertility of these mutant animals is still unknown, it is possible that the lack of epididymal CRISP4 is partially compensated by the presence of CRISP1, the other epididymal CRISP family member.

#### Crisp1^−/−^/Crisp4^−/−^

Both CRISP1 and CRISP4 are expressed in the epididymis in high concentrations (Eberspaecher et al., 1995; Krätzschmar et al., 1996), bind to sperm during epididymal transit and participate in sperm-ZP interaction and gamete fusion (Da Ros et al., 2008; Carvajal et al., 2018), supporting the idea that they could compensate for each other to ensure fertility success. This led to the generation of two different models of males lacking both CRISP1 and CRISP4 (Carvajal et al., 2018; Hu et al., 2018). While Hu et al. (2018) found no differences in fertility between mutant and control mice, concluding that epididymal CRISP are not absolutely required for male fertility, observations from our laboratory showed that *Crisp1* ^*−/−*^ */Crisp4* ^*−/−*^ colony exhibited a clear disruption of fertility (Carvajal et al., 2018). Differences in the genetic background and/or environmental conditions might explain the different phenotypes observed in the two studies. The impaired fertility of *Crisp1* ^*−/−*^ */Crisp4* ^*−/−*^ revealed, for the first time, the relevance of CRISP proteins for fertility and confirmed the existence of compensatory mechanisms among CRISP family members. In this way, whereas in *Crisp2* ^*−/−*^ */Crisp4* ^*−/−*^ mice, CRISP4 could be partially compensated by the presence of epididymal CRISP1, it is likely that the simultaneous lack of the two epididymal proteins in *Crisp1* ^*−/−*^ */Crisp4* ^*−/−*^, cannot be compensated by the remaining family members (i.e., CRISP2 and CRISP3).

Analysis of subfertile *Crisp1* ^*−/−*^ */Crisp4* ^*−/−*^ males showed they exhibited an immature epididymal epithelium and abnormal luminal acidification, contributing to a better understanding of the fine-tuning mechanisms underlying epididymal sperm maturation (Carvajal et al., 2018). Interestingly, fertility rates correlated with the percentages of fertilized oocytes recovered from the oviduct, supporting the idea that fertility impairment in these mutant mice are mainly due to *in vivo* fertilization defects. This idea was supported by the *in vitro* fertilization studies showing that *Crisp1* ^*−/−*^ */Crisp4* ^*−/−*^ mutant sperm exhibited a lower ability to fertilize oocytes either with or without their coats, consistent with the reported roles for CRISP1 and CRISP4 in sperm-ZP interaction and gamete fusion (Cohen et al., 2000b; Busso et al., 2007a; Carvajal et al., 2018). Subsequent studies showed that sperm fertilizing defects might be due to a failure of mutant sperm to undergo a normal capacitation process as judged by the clear alterations in capacitation-associated sperm parameters such as protein tyrosine phosphorylation, progesterone-induced acrosome reaction and hyperactivation (Carvajal et al., 2018; Hu et al., 2018). Considering the abnormal epididymal epithelium and luminal acidification, it is likely that sperm functional defects occur as a consequence of a defective sperm maturation process.

#### Crisp1^−/−^/Crisp3^−/−^

The *Crisp1* ^*−/−*^ */Crisp3* ^*−/−*^ colony was generated by CRISPR-Cas 9 technology (Wang et al., 2013; Curci et al., 2020). Analysis of animal fertility showed that these mutant males were subfertile, indicating that the absence of CRISP1 in combination with either CRISP4 or CRISP3 unveils the important role of CRISP proteins for optimal fertility. However, differently to what it was observed in the *Crisp1* ^*−/−*^ */Crisp4* ^*−/−*^ double knockout animals, normal levels of fertilization rates were observed when the eggs were recovered from the ampulla of control females mated with *Crisp1* ^*−/−*^ */Crisp3* ^*−/−*^ males (Curci et al., 2020), supporting that fertility inhibition in this colony occurred as a consequence of post-fertilization defects. Subsequent studies showed that male subfertility was associated, at least in part, with a failure of fertilized eggs to reach the blastocyst stage, revealing the relevance of CRISP1 and CRISP3 for early embryo development and supporting the impact of paternal factors in this process. It remains to be clarified whether impaired embryo development occurs as a consequence of a delayed *in vivo* fertilization. Interestingly in this regard, examination of ejaculated sperm within the uterus of control mated females showed that whereas control sperm were moving freely in the uterine fluid, *Crisp1* ^*−/−*^ */Crisp3* ^*−/−*^ sperm were mostly inmotile and forming aggregates within a viscous uterine fluid (Curci et al., 2020). This phenotype might be associated with an alteration of the reported ability of CRISP proteins to form amyloid-like structures (Sheng et al., 2019) known to trap damaged sperm within the uterus (Roan et al., 2017) and/or with coagulation/liquefaction defects within the uterus (Magdaleno et al., 1997; Udby et al., 2002) due to the lack of CRISP3 in the seminal plasma. These sperm motility defects within the uterus could be responsible for sperm transport deficiencies that, finally, lead to a delayed fertilization and embryo development defects in this colony. Nevertheless, we cannot exclude the possibility that embryo development deficiencies are due to sperm epididymal maturation and/or capacitation defects reported to affect embryonic development (Orgebin-Crist et al., 1967; Conine et al., 2018; Navarrete et al., 2019).

### Multiple (Triple and Quadruple) Knockout Models

The normal fertility and the subfertility of the different single and double knockout models support a functional compensation of the lacking proteins by the remaining members of the family that led to the generation of multiple knockout models lacking more than two *Crisp* genes simultaneously (Curci et al., 2020). Triple knockout (TKO) male mice lacking *Crisp1*, *Crisp2*, and *Crisp3* genes as well quadruple knockout (QKO) males deficient in the four CRISP members showed a dramatic inhibition in their fertility with an average of less than one born pup and a high proportion of sterile males (Curci et al., 2020) revealing, for the first time, the essential role of CRISP proteins for animal fertility. In this case, the severe fertility phenotype in TKO and QKO males was accompanied by significantly lower *in vivo* fertilization rates in the ampulla compared to controls which could be due to sperm transport and/or gamete interaction defects. Consistent with the fact that multiple knockout mice lack *Crisp1* and *Crisp3* genes, TKO and QKO ejaculated sperm were also mostly inmotile within the uterine fluid and trapped into aggregates in a very viscous fluid as previously described for the *Crisp1* ^*−/−*^/*Crisp3* ^*−/−*^ colony.

Besides motility defects at uterine level, sperm migration studies revealed that TKO and QKO sperm were capable of reaching the lower oviduct but exhibited clear defects in migrating within the organ (Curci et al., 2020). Although oviductal migration defects for *Crisp1* ^*−/−*^/*Crisp3* ^*−/−*^cannot be excluded, the normal *in vivo* fertilization rates observed for this colony does not favor this possibility. These observations support that oviductal migration defects in multiple knockout mice are likely due to the midpiece rigidity phenotype observed in ejaculated TKO and QKO (Lim et al., 2019; Curci et al., 2020) but not in *Crisp1* ^*−/−*^/*Crisp3* ^*−/−*^ sperm, associated with the lack of *Crisp2* and leading to defects in hyperactivation known to be required for detaching sperm from the isthmus epithelium.

In addition to these *in vivo* observations, *in vitro* fertilization studies revealed that TKO and QKO sperm had serious deficiencies in their ability to fertilize COC and ZP-intact eggs with no accumulation of sperm in the perivitelline space, indicating that fertilization failure could be attributed mostly to sperm defects to interact with the egg coats. This was further supported by the significantly lower levels of both hyperactivation and acrosome reaction observed in multiple mutant sperm. Hyperactivation failure could explain the impairment of both oviductal migration and egg coat penetration observed in multiple KO sperm (Curci et al., 2020). The finding that QKO but not TKO were unable to fertilize ZP-free eggs together with gamete fusion rates in TKO sperm that did not differ from those observed for *Crisp1^−/−^* or *Crisp2^−/−^* single knockout sperm argues against the involvement of CRISP3 in gamete fusion and reveals a key role of CRISP4 in this event supported by the low gamete fusion rates observed in those knockout models lacking *Crisp4* (i.e., *Crisp4^−/−^*, *Crisp1^−/−^/Crisp4^−/−^*, *Crisp2^−/−^/Crisp4^−/−^*) (Carvajal et al., 2018). Although the mechanisms underlying CRISP4 involvement in gamete fusion are still under investigation, they could be linked to CRISP4 interaction with egg complementary sites as observed for both CRISP1 (Rochwerger et al., 1992; Da Ros et al., 2008) and CRISP2 (Busso et al., 2007b) and/or the reported involvement of CRISP4 in the acrosome reaction (Gibbs et al., 2011; Turunen et al., 2012; Carvajal et al., 2018) known to be essential for gamete fusion (Yanagimachi, 1994). Interestingly, the finding that QKO males did not exhibit a stronger fertility phenotype compared to that observed for TKO males, supports the notion that *in vivo* fertilization failure is mainly due to sperm defects in those events that precede gamete fusion such as sperm migration within the oviduct and penetration of the egg coats.

Consistent with the ability of CRISP proteins to regulate Ca^2+^ channels and the relevance of this cation for most sperm functional events, QKO sperm did not show the characteristic intracellular Ca^2+^ increase that occurs during sperm capacitation, indicating that multiple mutant sperm exhibit a dysregulation of Ca^2+^ homeostasis that probably reflects the final balance of the individual contribution of each CRISP to Ca^2+^regulation. Finally, multiple mutant males exhibited clear embryo development defects as those previously described for the *Crisp1* ^*−/−*^/*Crisp3* ^*−/−*^colony (Curci el al., 2020).

Collectively, analysis of the different phenotypes observed in single, double and multiple knockout models showed that the subfertility of *Crisp1* and *Crisp3* males is associated with both the presence of inmotile sperm within the uterus and embryo development failure. As expected, these defects were also observed in infertile TKO and QKO males lacking *Crisp1* and *Crisp3.* However, besides these deficiencies, TKO and QKO mice exhibit sperm with midpiece rigidity due to the additional lack of testicular CRISP2 which affects hyperactivation and, thus, both oviductal migration and gamete interaction due to the critical role of this vigorous motility for detachment of sperm from the isthmus and egg coat penetration (Yanagimachi, 1994; Florman and Fissore, 2015). Finally, the few TKO and QKO sperm that might reach the ampulla exhibit severe gamete interaction defects due to egg coat penetration deficiencies generated by the lack of *Crisp2* and both sperm-ZP binding and gamete fusion failure associated with the lack of epididymal *Crisp1* and *Crisp4* genes.

The finding that mice lacking three or four CRISP proteins exhibit more severe phenotypes than single or double CRISP knockout mice supports the idea that the combined mutations of *Crisp* members lead to disruption of multiple independent pathways. Similar results were observed for the sperm β-defensin protein family since animals were fertile when lacking one individual gene, subfertile when lacking two or three genes (Zhang et al., 2018) and completely infertile when nulling the whole family member expression (Zhou et al., 2013).

## Conclusion and Perspectives

Biochemical, cellular and genetic approaches revealed that CRISP proteins play key functional roles in the successive stages of the fertilization process (i.e., cumulus penetration, sperm-ZP binding, ZP penetration, gamete fusion) through different mechanisms that include ligand-receptor interactions as well as regulation of several capacitation-associated events (i.e., acrosome reaction, hyperactivation, etc.), likely through their ability to regulate different Ca^2+^ channels. Moreover, results showed that CRISP are involved not only in gamete interaction but also in previous and subsequent steps such as sperm migration within the female tract and early embryo development. Collectively, it can be concluded that CRISP proteins are essential for male fertility due to a combination of different functional roles during the fertilization process. These findings also support the use of CRISP proteins for contraception, opening the possibility of targeting CRISP activity at different levels.

**FIGURE 3:**
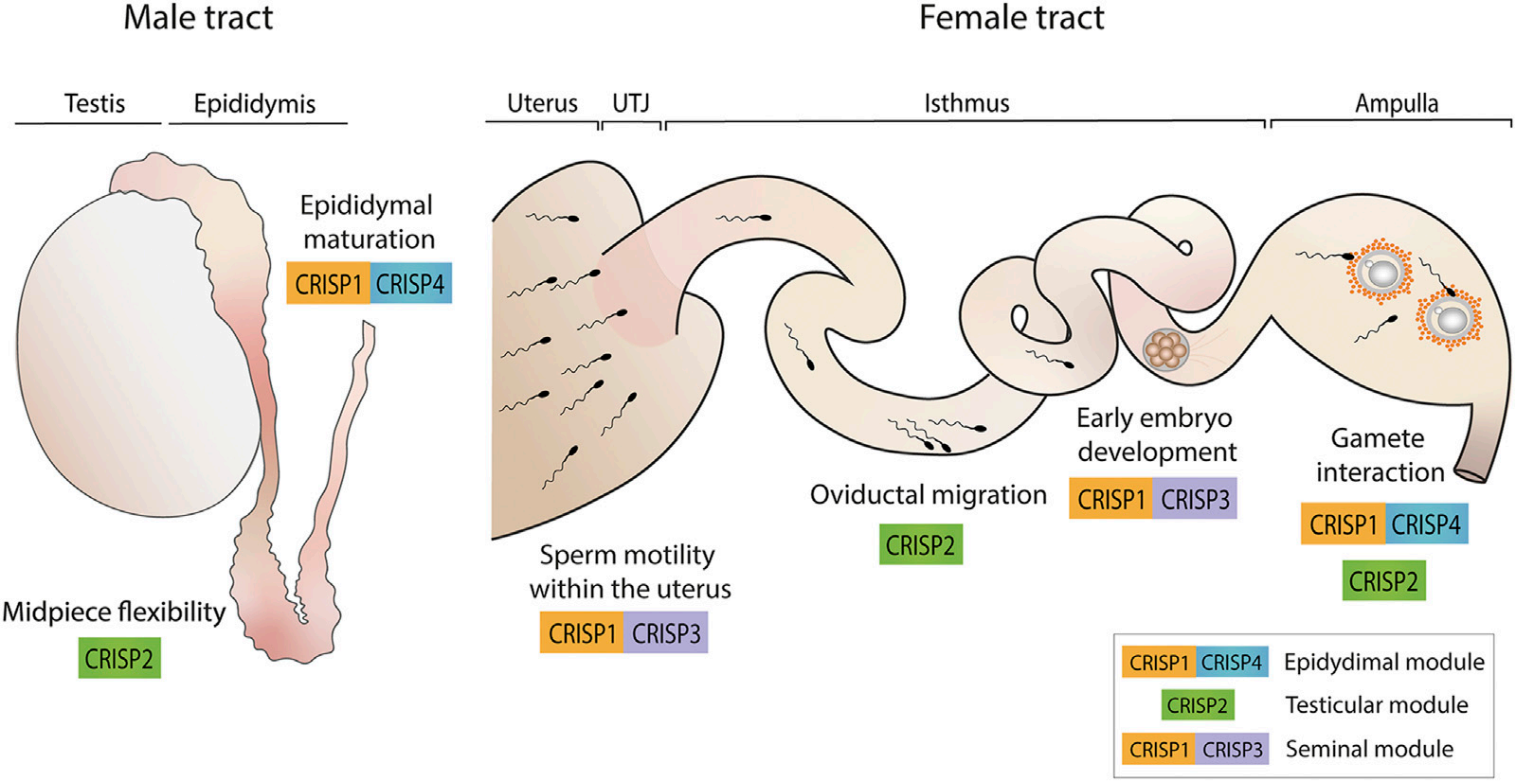
CRISP functional modules and their roles in male and female reproductive tracts. Epididymal CRISP1 and CRISP4 (epididymal module) were found to be involved in sperm maturation as well as in gamete interaction. Testicular CRISP2 (testicular module) was reported to be required for the development of sperm midpiece flexibility critical for hyperactivation and, thus, for oviductal sperm migration and gamete interaction. Finally, CRISP1 and CRISP3 (seminal module) were found to be essential for maintaining sperm motility within the uterus and for early embryo development. A schematic sperm representation is included.

Evidence supports the idea that CRISP proteins have evolved to perform redundant as well as specialized functions to ensure fertility success and are organized in functional modules within the family that work through independent pathways and contribute distinctly to fertility success (Figure 3). In this way, whereas epididymal CRISP1 and CRISP4 (epididymal module) are critical for epididymal sperm maturation, play similar roles in gamete interaction and can compensate for each other, testicular CRISP2 and seminal CRISP3 appear to have specific functions not compensated by the remaining CRISP family members. Whereas CRISP2 (testicular module) showed to be critical for the development of sperm midpiece flexibility that occurs during epididymal maturation and, thus, for oviductal sperm migration and egg coat penetration, CRISP3, together with CRISP1 (seminal module), seems to be needed for maintaining sperm motility within the uterus as well as for early embryo development.

Redundancy and compensatory mechanisms like those observed within the CRISP protein family are particularly important in the case of spermatozoa which are transcriptionally and translationally inactive cells and carry several protein families (i.e., ADAMs, defensins, CRISP, etc.) to guarantee their functionality. This emphasizes the importance of generating multiple knockout of family genes since single knockout models might be masking the true functional relevance of family proteins.

Considering the high sequence and functional homology between rodent and human CRISP proteins, it is likely that the same redundant and specific mechanisms operate within the human CRISP family giving rise to the same functional modules (i.e., epididymal CRISP1, testicular CRISP2 and seminal CRISP3). Furthermore, as the three hCRISP genes are located very close to each other within the same chromosome, it is possible that a single rearrangement in that region is responsible for some cases of unexplained infertility due to the simultaneous absence of more than one human CRISP protein, supporting the relevance of our observations for the diagnosis and treatment of human infertility as well as for the development of new non-hormonal contraceptive options.

## Abbreviations

CatSper, cation channel of sperm; COC, cumulus-oocyte complexes; CRISP, Cysteine Rich Secretory Proteins; QKO, quadruple knockout; TKO, triple knockout; TRPM8, transient receptor potential membrane 8; RyR, ryanodine receptor; ZP, zona pellucida.

## References

[B1] Arévalo L. Brukman N. G. Cuasnicú P. S. Roldan E. R. S. (2020). Evolutionary Analysis of Genes Coding for Cysteine-RIch Secretory Proteins (CRISPs) in Mammals. BMC Evol. Biol. 20, 67. 10.1186/s12862-020-01632-5 32513118 PMC7278046

[B2] Austin C. R. (1952). The 'Capacitation' of the Mammalian Sperm. Nature 170, 326. 10.1038/170326a0 12993150

[B3] Belardin L. Camargo M. Intasqui P. Antoniassi M. Fraietta R. Bertolla R. (2019). Cysteine-rich Secretory Protein 3: Inflammation Role in Adult Varicocoele. Andrology 7, 53–61. 10.1111/andr.12555 30354034

[B4] Bjartell A. S. Al-Ahmadie H. Serio A. M. Eastham J. A. Eggener S. E. Fine S. W. (2007). Association of Cysteine-Rich Secretory Protein 3 and Beta-Microseminoprotein with Outcome after Radical Prostatectomy. Clin. Cancer Res. 13, 4130–4138. 10.1158/1078-0432.CCR-06-3031 17634540 PMC2660867

[B5] Borges B. C. Garcia-Galiano D. da Silveira Cruz-Machado S. Han X. Gavrilina G. B. Saunders T. L. (2017). Obesity-Induced Infertility in Male Mice Is Associated with Disruption of Crisp4 Expression and Sperm Fertilization Capacity. Endocrinology 158, 2930–2943. 10.1210/en.2017-00295 28911169 PMC5659670

[B6] Brukman N. G. Miyata H. Torres P. Lombardo D. Caramelo J. J. Ikawa M. (2016). Fertilization Defects in Sperm from Cysteine-Rich Secretory Protein 2 (Crisp2) Knockout Mice: Implications for Fertility Disorders. Mol. Hum. Reprod. 22, 240–251. 10.1093/molehr/gaw005 26786179

[B7] Busso D. Cohen D. J. Hayashi M. Kasahara M. Cuasnicú P. S. (2005). Human Testicular Protein TPX1/CRISP-2: Localization in Spermatozoa, Fate after Capacitation and Relevance for Gamete Interaction. Mol. Hum. Reprod. 11, 299–305. 10.1093/molehr/gah156 15734896

[B8] Busso D. Cohen D. J. Maldera J. A. Dematteis A. Cuasnicu P. S. (2007). A Novel Function for CRISP1 in Rodent Fertilization: Involvement in Sperm-Zona Pellucida Interaction. Biol. Reprod. 77, 848–854. 10.1095/biolreprod.107.061788 17671267

[B9] Busso D. Goldweic N. M. Hayashi M. Kasahara M. Cuasnicú P. S. (2007). Evidence for the Involvement of Testicular Protein CRISP2 in Mouse Sperm-Egg Fusion. Biol. Reprod. 76, 701–708. 10.1095/biolreprod.106.056770 17202389

[B10] Cameo M. S. Blaquier J. A. (1976). Androgen-controlled Specific Proteins in Rat Epididymis. J. Endocrinol. 69, 47–55. 10.1677/joe.0.0690047 1270959

[B11] Carvajal G. Brukman N. G. Weigel Muñoz M. Battistone M. A. Guazzone V. A. Ikawa M. (2018). Impaired Male Fertility and Abnormal Epididymal Epithelium Differentiation in Mice Lacking CRISP1 and CRISP4. Sci. Rep. 8, 17531. 10.1038/s41598-018-35719-3 30510210 PMC6277452

[B12] Chang H. Suarez S. S. (2011). Two Distinct Ca(2+) Signaling Pathways Modulate Sperm Flagellar Beating Patterns in Mice. Biol. Reprod. 85, 296–305. 10.1095/biolreprod.110.089789 21389347 PMC3142258

[B13] Chang M. C. (1951). Fertilizing Capacity of Spermatozoa Deposited into the Fallopian Tubes. Nature 168, 697–698. 10.1038/168697b0 14882325

[B14] Cohen D. J. Ellerman D. A. Busso D. Morgenfeld M. M. Piazza A. D. Hayashi M. (2001). Evidence that Human Epididymal Protein ARP Plays a Role in Gamete Fusion through Complementary Sites on the Surface of the Human Egg. Biol. Reprod. 65, 1000–1005. 10.1095/biolreprod65.4.1000 11566719

[B15] Cohen D. J. Ellerman D. A. Cuasnicú P. S. (2000). Mammalian Sperm-Egg Fusion: Evidence that Epididymal Protein DE Plays a Role in Mouse Gamete Fusion. Biol. Reprod. 63, 462–468. 10.1095/biolreprod63.2.462 10906051

[B16] Cohen D. J. Rochwerger L. Ellerman D. A. Morgenfeld M. M. Busso D. Cuasnicú P. S. (2000). Relationship between the Association of Rat Epididymal Protein "DE" with Spermatozoa and the Behavior and Function of the Protein. Mol. Reprod. Dev. 56, 180–188. 10.1002/(SICI)1098-2795(200006)56:2<180:AID-MRD9>3.0.CO;2-4 10813850

[B17] Conine C. C. Sun F. Song L. Rivera-Pérez J. A. Rando O. J. (2018). Small RNAs Gained during Epididymal Transit of Sperm Are Essential for Embryonic Development in Mice. Dev. Cel 46, 470–e3. 10.1016/j.devcel.2018.06.024 PMC6103825 30057276

[B18] Cuasnicu P. S. Da Ros V. G. Weigel Muñoz M. Cohen D. J. (2016). “Acrosome Reaction as a Preparation for Gamete Fusion,”. Sperm Acrosome Biogenesis and Function during Fertilization, Advances in Anatomy, Embryology and Cell Biology. Editor Buffone M. G. (Springer), 220. Chapter 9. 10.1007/978-3-319-30567-7_9 27194355

[B19] Cuasnicú P. S. Conesa D. Rochwerger L. (1990). “Potential Contraceptive Use of an Epididymal Protein that Participates in Fertilization,” in Gamete Interaction. Prospects for Immunocontraception. Editors J Alexander N. Griffin D. M Spieler J. M Waites G. (New York): Wiley-Liss), 143–153.

[B20] Curci L. Brukman N. G. Weigel Muñoz M. Rojo D. Carvajal G. Sulzyk V. (2020). Functional Redundancy and Compensation: Deletion of Multiple Murine Crisp Genes Reveals Their Essential Role for Male Fertility. FASEB J. 34, 15718–15733. 10.1096/fj.202001406r 33037689

[B21] Da Ros V. G. Maldera J. A. Willis W. D. Cohen D. J. Gelman D. M. Rubinstein M. (2008). Impaired Sperm Fertilizing Ability in Mice Lacking Cysteine-RIch Secretory Protein 1 (CRISP1). Dev. Biol. 320, 12–18. 10.1016/j.ydbio.2008.03.015 18571638 PMC2603034

[B22] Da Ros V. G. Muñoz M. W. Battistone M. A. Brukman N. G. Carvajal G. Curci L. (2015). From the Epididymis to the Egg: Participation of CRISP Proteins in Mammalian Fertilization. Asian J. Androl. 17, 711–715. 10.4103/1008-682x.155769 26112483 PMC4577577

[B23] Doty A. Buhi W. C. Benson S. Scoggin K. E. Pozor M. Macpherson M. (2011). Equine CRISP3 Modulates Interaction between Spermatozoa and Polymorphonuclear Neutrophils. Biol. Reprod. 85, 157–164. 10.1095/biolreprod.110.084491 21389342

[B24] Du Y. Huang X. Li J. Hu Y. Zhou Z. Sha J. (2006). Human Testis Specific Protein 1 Expression in Human Spermatogenesis and Involvement in the Pathogenesis of Male Infertility. Fertil. Steril 85, 1852–1854. 10.1016/j.fertnstert.2005.11.064 16759931

[B25] Eberspaecher U. Roosterman D. Krätzschmar J. Haendler B. Habenicht U. F. Becker A. (1995). Mouse Androgen-dependent Epididymal Glycoprotein CRISP-1 (DE/AEG): Isolation, Biochemical Characterization, and Expression in Recombinant Form. Mol. Reprod. Dev. 42, 157–172. 10.1002/mrd.1080420205 8562061

[B26] Ellerman D. A. Brantúa V. S. Martínez S. P. Cohen D. J. Conesa D. Cuasnicú P. S. (1998). Potential Contraceptive Use of Epididymal Proteins: Immunization of Male Rats with Epididymal Protein DE Inhibits Sperm Fusion Ability. Biol. Reprod. 59, 1029–1036. 10.1095/biolreprod59.5.1029 9780306

[B27] Ellerman D. A. Busso D. Maldera J. A. Cuasnicú P. S. (2008). Immunocontraceptive Properties of Recombinant Sperm Protein DE: Implications for the Development of Novel Contraceptives. Fertil. Steril 89, 199–205. 10.1016/j.fertnstert.2007.02.025 17482178

[B28] Ellerman D. A. Cohen D. J. Weigel Muñoz M. Da Ros V. G. Ernesto J. I. Tollner T. L. (2010). Immunologic Behavior of Human Cysteine-Rich Secretory Protein 1 (hCRISP1) in Primates: Prospects for Immunocontraception. Fertil. Steril 93, 2551–2556. 10.1016/j.fertnstert.2010.01.075 20226442

[B29] Ellerman D. E. Cohen D. J. Da Ros V. G. Morgenfeld M. M. Busso D. Cuasnicú P. S. (2006). Sperm Protein “DE” Mediates Gamete Fusion through an Evolutionarily Conserved Site of the CRISP Family. Dev. Biol. 297, 228–237. 10.1016/j.ydbio.2006.05.013 16872593

[B30] Enger T. B. Aure M. H. Jensen J. L. Galtung H. K. (2014). Calcium Signaling and Cell Volume Regulation Are Altered in Sjögren's Syndrome. Acta Odontol Scand. 72, 549–556. 10.3109/00016357.2013.879995 24471729

[B31] Ernesto J. I. Weigel Muñoz M. Battistone M. A. Vasen G. Martínez-López P. Orta G. (2015). CRISP1 as a Novel CatSper Regulator that Modulates Sperm Motility and Orientation during Fertilization. J. Cel Biol 210, 1213–1224. 10.1083/jcb.201412041 PMC4586743 26416967

[B32] Evans J. D'Sylva R. Volpert M. Jamsai D. Merriner D. J. Nie G. (2015). Endometrial CRISP3 Is Regulated throughout the Mouse Estrous and Human Menstrual Cycle and Facilitates Adhesion and Proliferation of Endometrial Epithelial Cells. Biol. Reprod. 92, 99. 10.1095/biolreprod.114.127480 25715794

[B33] Florman H. M. Fissore R. A. (2015). “Fertilization in Mammals,” in Knobil and Neill’s Physiology of Reproduction. Editors M Plant T. J Zeleznik A. (Massachusetts): Elsevier/Academic Press), 149–196.

[B34] Gaikwad A. S. Nandagiri A. Potter D. L. Nosrati R. O'Connor A. E. Jadhav S. (2021). CRISPs Function to Boost Sperm Power Output and Motility. Front Cel Dev Biol 9, 693258. 10.3389/fcell.2021.693258 PMC8374954 34422816

[B35] Gao F. Wang P. Wang K. Fan Y. Chen Y. Chen Y. (2021). Investigation into the Relationship between Sperm Cysteine-Rich Secretory Protein 2 (CRISP2) and Sperm Fertilizing Ability and Fertility of Boars. Front. Vet. Sci. 8, 653413. 10.3389/fvets.2021.653413 33996980 PMC8119884

[B36] Garberi J. C. Fontana J. D. Blaquier J. A. (1982). Carbohydrate Composition of Specific Rat Epididymal Protein. Int. J. Androl. 5, 619–626. 10.1111/j.1365-2605.1982.tb00296.x 7160923

[B37] Gholami D. Salman Yazdi R. Jami M. S. Ghasemi S. Sadighi Gilani M. A. Sadeghinia S. (2020). The Expression of Cysteine-Rich Secretory Protein 2 (CRISP2) and miR-582-5p in Seminal Plasma Fluid and Spermatozoa of Infertile Men. Gene 730, 144261. 10.1016/j.gene.2019.144261 31778754

[B38] Gibbs G. M. O'Bryan M. K. (2007). Cysteine Rich Secretory Proteins in Reproduction and Venom. Soc. Reprod. Fertil. Suppl. 65, 261–267. 17644967

[B39] Gibbs G. M. Orta G. Reddy T. Koppers A. J. Martínez-López P. de la Vega-Beltràn J. L. (2011). Cysteine-rich Secretory Protein 4 Is an Inhibitor of Transient Receptor Potential M8 with a Role in Establishing Sperm Function. Proc. Natl. Acad. Sci. U S A. 108, 7034–7039. 10.1073/pnas.1015935108 21482758 PMC3084142

[B40] Gibbs G. M. Roelants K. O'Bryan M. K. (2008). The CAP Superfamily: Cysteine-Rich Secretory Proteins, Antigen 5, and Pathogenesis-Related 1 Proteins-Rroles in Reproduction, Cancer, and Immune Defense. Endocr. Rev. 29, 865–897. 10.1210/er.2008-0032 18824526

[B41] Gibbs G. M. Scanlon M. J. Swarbrick J. Curtis S. Gallant E. Dulhunty A. F. (2006). The Cysteine-Rich Secretory Protein Domain of Tpx-1 Is Related to Ion Channel Toxins and Regulates Ryanodine Receptor Ca2+ Signaling. J. Biol. Chem. 281, 4156–4163. 10.1074/jbc.M506849200 16339766

[B42] Giese A. Jude R. Kuiper H. Raudsepp T. Piumi F. Schambony A. (2002). Molecular Characterization of the Equine Testis-specific Protein 1 (TPX1) and Acidic Epididymal Glycoprotein 2 (AEG2) Genes Encoding Members of the Cysteine-Rich Secretory Protein (CRISP) Family. Gene 299, 101–109. 10.1016/s0378-1119(02)01018-1 12459257

[B43] Grande G. Vincenzoni F. Milardi D. Pompa G. Ricciardi D. Fruscella E. (2017). Cervical Mucus Proteome in Endometriosis. Clin. Proteomics 14, 7. 10.1186/s12014-017-9142-4 28174513 PMC5290661

[B44] Guo M. Teng M. Niu L. Liu Q. Huang Q. Hao Q. (2005). Crystal Structure of the Cysteine-Rich Secretory Protein Stecrisp Reveals that the Cysteine-Rich Domain Has a K+ Channel Inhibitor-like Fold. J. Biol. Chem. 280, 12405–12412. 10.1074/jbc.M413566200 15596436

[B45] Haendler B. Habenicht U. F. Schwidetzky U. Schüttke I. Schleuning W. D. (1997). Differential Androgen Regulation of the Murine Genes for Cysteine-Rich Secretory Proteins (CRISP). Eur. J. Biochem. 250, 440–446. 10.1111/j.1432-1033.1997.0440a.x 9428696

[B46] Haendler B. Krätzschmar J. Theuring F. Schleuning W. D. (1993). Transcripts for Cysteine-Rich Secretory Protein-1 (CRISP-1; DE/AEG) and the Novel Related CRISP-3 Are Expressed under Androgen Control in the Mouse Salivary Gland. Endocrinology 133, 192–198. 10.1210/endo.133.1.8319566 8319566

[B47] Hamann H. Jude R. Sieme H. Mertens U. Töpfer-Petersen E. Distl O. (2007). A Polymorphism within the Equine CRISP3 Gene Is Associated with Stallion Fertility in Hanoverian Warmblood Horses. Anim. Genet. 38, 259–264. 10.1111/j.1365-2052.2007.01594.x 17433013

[B48] Hardy D. M. Huang T. T. Driscoll W. J. Tung K. K. Wild G. C. (1988). Purification and Characterization of the Primary Acrosomal Autoantigen of guinea Pig Epididymal Spermatozoa. Biol. Reprod. 38, 423–437. 10.1095/biolreprod38.2.423 3282555

[B49] Harper C. V. Barratt C. L. Publicover S. J. (2004). Stimulation of Human Spermatozoa with Progesterone Gradients to Simulate Approach to the Oocyte. Induction of [Ca(2+)](i) Oscillations and Cyclical Transitions in Flagellar Beating. J. Biol. Chem. 279, 46315–46325. 10.1074/jbc.M401194200 15322137

[B50] Hayashi M. Fujimoto S. Takano H. Ushiki T. Abe K. Ishikura H. (1996). Characterization of a Human Glycoprotein with a Potential Role in Sperm-Egg Fusion: cDNA Cloning, Immunohistochemical Localization, and Chromosomal Assignment of the Gene (AEGL1). Genomics 32, 367–374. 10.1006/geno.1996.0131 8838800

[B51] Heidary Z. Zaki-Dizaji M. Saliminejad K. Khorramkhorshid H. R. (2019). Expression Analysis of the CRISP2, CATSPER1, PATE1 and SEMG1 in the Sperm of Men with Idiopathic Asthenozoospermia. J. Reprod. Infertil. 20, 70–75. 31058050 PMC6486568

[B52] Hu J. Merriner D. J. O'Connor A. E. Houston B. J. Furic L. Hedger M. P. (2018). Epididymal Cysteine-Rich Secretory Proteins Are Required for Epididymal Sperm Maturation and Optimal Sperm Function. Mol. Hum. Reprod. 24, 111–122. 10.1093/molehr/gay001 29361143

[B53] Jalkanen J. Huhtaniemi I. Poutanen M. (2005). Mouse Cysteine-Rich Secretory Protein 4 (CRISP4): a Member of the Crisp Family Exclusively Expressed in the Epididymis in an Androgen-dependent Manner. Biol. Reprod. 72, 1268–1274. 10.1095/biolreprod.104.035758 15673606

[B54] Jing X. Xing R. Zhou Q. Yu Q. Guo W. Chen S. (2011). Expressions of Cysteine-Rich Secretory Protein 2 in Asthenospermia. Zhonghua Nan Ke Xue 17, 203–207. 21485539

[B55] Judd J. E. Berndtson W. E. Castro A. C. (1997). Extragonadal Sperm Reserves, Sperm-Depletion Rates, Numbers of Sperm Per Mating, and Fertility with Successive Matings by Intact or Unilaterally Vasectomized Rats. J. Androl. 18, 698–707. 9432143

[B56] Kasahara M. Gutknecht J. Brew K. Spurr N. Goodfellow P. N. (1989). Cloning and Mapping of a Testis-specific Gene with Sequence Similarity to a Sperm-Coating Glycoprotein Gene. Genomics 5, 527–534. 10.1016/0888-7543(89)90019-0 2613236

[B57] Kjeldsen L. Cowland J. B. Johnsen A. H. Borregaard N. (1996). SGP28, a Novel Matrix Glycoprotein in Specific Granules of Human Neutrophils with Similarity to a Human Testis-specific Gene Product and a Rodent Sperm-Coating Glycoprotein. FEBS Lett. 380, 246–250. 10.1016/0014-5793(96)00030-0 8601434

[B58] Kohane A. C. Cameo M. S. Piñeiro L. Garberi J. C. Blaquier J. A. (1980). Distribution and Site of Production of Specific Proteins in the Rat Epididymis. Biol. Reprod. 23, 181–187. 10.1095/biolreprod23.1.181 7417663

[B59] Kohane A. C. González Echeverría F. M. Piñeiro L. Blaquier J. A. (1980). Interaction of Proteins of Epididymal Origin with Spermatozoa. Biol. Reprod. 23, 737–742. 10.1095/biolreprod23.4.737 7448274

[B60] Konttinen Y. T. Porola P. Konttinen L. Laine M. Poduval P. (2006). Immunohistopathology of Sjögren's Syndrome. Autoimmun. Rev. 6, 16–20. 10.1016/j.autrev.2006.03.003 17110311

[B61] Kosari F. Asmann Y. W. Cheville J. C. Vasmatzis G. (2002). Cysteine-rich Secretory Protein-3: a Potential Biomarker for Prostate Cancer. Cancer Epidemiol. Biomarkers Prev. 11, 1419–1426. 12433721

[B62] Krätzschmar J. Haendler B. Eberspaecher U. Roosterman D. Donner P. Schleuning W. D. (1996). The Human Cysteine-Rich Secretory Protein (CRISP) Family. Primary Structure and Tissue Distribution of CRISP-1, CRISP-2 and CRISP-3. Eur. J. Biochem. 236, 827–836. 10.1111/j.1432-1033.1996.t01-1-00827.x 8665901

[B63] Laine M. Porola P. Udby L. Kjeldsen L. Cowland J. B. Borregaard N. (2007). Low Salivary Dehydroepiandrosterone and Androgen-Regulated Cysteine-Rich Secretory Protein 3 Levels in Sjögren’s Syndrome. Arthritis Rheum. 56, 2575–2584. 10.1002/art.22828 17665393

[B64] Lim S. Kierzek M. O'Connor A. E. Brenker C. Merriner D. J. Okuda H. (2019). CRISP2 Is a Regulator of Multiple Aspects of Sperm Function and Male Fertility. Endocrinology 160, 915–924. 10.1210/en.2018-01076 30759213

[B65] Luo J. Yang J. Cheng Y. Li W. Yin T. L. Xu W. M. (2012). Immunogenicity Study of Plasmid DNA Encoding Mouse Cysteine-Rich Secretory Protein-1 (mCRISP1) as a Contraceptive Vaccine. Am. J. Reprod. Immunol. 68, 47–55. 10.1111/j.1600-0897.2012.01117.x 22429321

[B66] Luo J. Liu X. L. Zhang Y. Wang Y. Q. Xu W. M. Yang J. (2016). The Immunogenicity of CRISP1 Plasmid-Based Contraceptive Vaccine Can Be Improved when Using Chitosan Nanoparticles as the Carrier. Am.J.Reprod.Immunol. 75, 643–653. 10.1111/aji.12513 27105782

[B67] Maeda T. Nishida J. Nakanishi Y. (1999). Expression Pattern, Subcellular Localization and Structure-Ffunction Relationship of Rat Tpx-1, a Spermatogenic Cell Adhesion Molecule Responsible for Association with Sertoli Cells. Dev. Growth Differ. 41, 715–722. 10.1046/j.1440-169x.1999.00470.x 10646801

[B68] Maeda T. Sakashita M. Ohba Y. Nakanishi Y. (1998). Molecular Cloning of the Rat Tpx-1 Responsible for the Interaction between Spermatogenic and Sertoli Cells. Biochem. Biophys. Res. Commun. 248, 140–146. 10.1006/bbrc.1998.8918 9675100

[B69] Magdaleno L. Gasset M. Varea J. Schambony A. M. Urbanke C. Raida M. (1997). Biochemical and Conformational Characterisation of HSP-3, a Stallion Seminal Plasma Protein of the Cysteine-Rich Secretory Protein (CRISP) Family. FEBS Lett. 420, 179–185. 10.1016/s0014-5793(97)01514-7 9459306

[B70] Maldera J. A. Weigel Muñoz M. Chirinos M. Busso D. G E Raffo F. Battistone M. A. (2014). Human Fertilization: Epididymal hCRISP1 Mediates Sperm-Zona Pellucida Binding through its Interaction with ZP3. Mol. Hum. Reprod. 20, 341–349. 10.1093/molehr/gat092 24334245

[B71] Martínez-López P. Treviño C. L. De la Vega-Beltrán J. L. De Blas G. Monroy E. Beltrán C. (2010). TRPM8 in Mouse Sperm Detects Temperature Changes and May Influence the Acrosome Reaction. J. Cel Physiol. 226, 1620–1631. 10.1002/jcp.22493 21413020

[B72] Miyata H. Castaneda J. M. Fujihara Y. Yu Z. Archambeault D. R. Isotani A. (2016). Genome Engineering Uncovers 54 Evolutionarily Conserved and Testis-Enriched Genes that Are Not Required for Male Fertility in Mice. Proc. Natl. Acad. Sci. U S A. 113, 7704–7710. 10.1073/pnas.1608458113 27357688 PMC4948324

[B73] Mizuki N. Sarapata D. E. Garcia-Sanz J. A. Kasahara M. (1992). The Mouse Male Germ Cell-specific Gene Tpx-1: Molecular Structure, Mode of Expression in Spermatogenesis, and Sequence Similarity to Two Non-mammalian Genes. Mamm. Genome 3, 274–280. 10.1007/BF00292155 1638086

[B74] Mochca-Morales J. Martin B. M. Possani L. D. (1990). Isolation and Characterization of Helothermine, a Novel Toxin from *Heloderma horridum* Horridum (Mexican Beaded Lizard) Venom. Toxicon 28, 299–309. 10.1016/0041-0101(90)90065-f 1693019

[B75] Muñoz M. W. Ernesto J. I. Bluguermann C. Busso D. Battistone M. A. Cohen D. J. (2012). Evaluation of Testicular Sperm CRISP2 as a Potential Target for Contraception. J. Androl. 33, 1360–1370. 10.2164/jandrol.112.016725 22653965

[B76] Nandagiri A. Gaikwad A. S. Potter D. L. Nosrati R. Soria J. O'Bryan M. K. (2021). Flagellar Energetics from High-Resolution Imaging of Beating Patterns in Tethered Mouse Sperm. Elife 10, e62524. 10.7554/elife.62524 33929317 PMC8159377

[B77] Navarrete F. A. Aguila L. Martin-Hidalgo D. Tourzani D. A. Luque G. M. Ardestani G. (2019). Transient Sperm Starvation Improves the Outcome of Assisted Reproductive Technologies. Front. Cel Dev Biol 7, 262. 10.3389/fcell.2019.00262 PMC6848031 31750304

[B78] Nimlamool W. Bean B. S. Lowe-Krentz L. J. (2013). Human Sperm CRISP2 Is Released from the Acrosome during the Acrosome Reaction and Re-associates at the Equatorial Segment. Mol. Reprod. Dev. 80, 488–502. 10.1002/mrd.22189 23661501

[B79] Noh B. J. Sung J. Y. Kim Y. W. Chang S. G. Park Y. K. (2016). Prognostic Value of ERG, PTEN, CRISP3 and SPINK1 in Predicting Biochemical Recurrence in Prostate Cancer. Oncol. Lett. 11, 3621–3630. 10.3892/ol.2016.4459 27284364 PMC4887942

[B80] Nolan M. A. Wu L. Bang H. J. Jelinsky S. A. Roberts K. P. Turner T. T. (2006). Identification of Rat Cysteine-Rich Secretory Protein 4 (Crisp4) as the Ortholog to Human CRISP1 and Mouse Crisp4. Biol. Reprod. 74, 984–991. 10.1095/biolreprod.105.048298 16467491

[B81] O’Bryan M. K. Loveland K. L. Herszfeld D. McFarlane J. R. Hearn M. T. De Kretser D. M. (1998). Identification of a Rat Testis–specific Gene Encoding a Potential Rat Outer Dense Fibre Protein. Mol. Reprod. Dev. 50, 313–322. 10.1002/(sici)1098-2795(199807)50:3<313:aid-mrd7>3.0.co;2-m 9621307

[B82] O’Bryan M. K. Sebire K. Meinhardt A. Edgar K. Keah H. H. Hearn M. T. W. (2001). Tpx-1 Is a Component of the Outer Dense Fibers and Acrosome of Rat Spermatozoa. Mol. Rep. Dev. 58, 116–125. 10.1002/1098-2795(200101)58:1<116:aid-mrd14>3.0.co;2-8 11144214

[B83] Orgebin-Crist M-C. Brantley E. B. Hart J. R. (1967). Maturation of Spermatozoa in the Rabbit Epididymis: Fertilizing Ability and Embryonic Mortality in Does Inseminated with Epididymal Spermatozoa. Ann. Biol. Anim. Biochim. Biophys. 7, 373–389. 10.1051/rnd:19670403

[B84] Perez Martinez S. Conesa D. Cuasnicú P. S. (1995). Potential Contraceptive Use of Epididymal Proteins: Evidence for the Participation of Specific Antibodies against Rat Epididymal Protein DE in Male and Female Fertility Inhibition. J. Reprod. Immunol. 29, 31–45. 10.1016/0165-0378(95)00927-d 8531190

[B85] Reddy T. Gibbs G. M. Merriner D. J. Kerr J. B. O'Bryan M. K. (2008). Cysteine-rich Secretory Proteins Are Not Exclusively Expressed in the Male Reproductive Tract. Dev. Dyn. 237, 3313–3323. 10.1002/dvdy.21738 18924239

[B86] Ren D. Navarro B. Perez G. Jackson A. C. Hsu S. Shi Q. (2001). A Sperm Ion Channel Required for Sperm Motility and Male Fertility. Nature 413, 603–609. 10.1038/35098027 11595941 PMC8462998

[B87] Rennhack A. Schiffer C. Brenker C. Fridman D. Nitao E. T. Cheng Y. M. (2018). A Novel Cross-Species Inhibitor to Study the Function of CatSper Ca2+ Channels in Sperm. Br. J. Pharmacol. 175, 3144–3161. 10.1111/bph.14355 29723408 PMC6031884

[B88] Roan N. R. Sandi-Monroy N. Kohgadai N. Usmani S. M. Hamil K. G. Neidleman J. (2017). Semen Amyloids Participate in Spermatozoa Selection and Clearance. Elife 6, e24888. 10.7554/eLife.24888 28653619 PMC5487211

[B89] Robaire B. Hinton B. T. (2015). “The Epididymis,” in Knobil and Neill’s Physiology of Reproduction*:* Two-Volume Set. Editors Zeleznik A. J. Plant T. M.. 4th Edn (Amsterdam: Elsevier), Vol. 1, 691–771.

[B90] Roberts K. P. Wamstad J. A. Ensrud K. M. Hamilton D. W. (2003). Inhibition of Capacitation-Associated Tyrosine Phosphorylation Signaling in Rat Sperm by Epididymal Protein Crisp-1. Biol. Reprod. 69, 572–581. 10.1095/biolreprod.102.013771 12700197

[B91] Rochwerger L. Cohen D. J. Cuasnicú P. S. (1992). Mammalian Sperm-Egg Fusion: the Rat Egg Has Complementary Sites for a Sperm Protein that Mediates Gamete Fusion. Dev. Biol. 153, 83–90. 10.1016/0012-1606(92)90093-v 1516754

[B92] Schambony A. Gentzel M. Wolfes H. Raida M. Neumann U. Töpfer-Petersen E. (1998). Equine CRISP-3: Primary Structure and Expression in the Male Genital Tract. Biochim. Biophys. Acta 1387, 206–216. 10.1016/s0167-4838(98)00122-8 9748582

[B93] Schwidetzky U. Haendler B. Schleuning W. D. (1995). Isolation and Characterization of the Androgen-dependent Mouse Cysteine-Rich Secretory Protein-3 (CRISP-3) Gene. Biochem. J. 309 ( Pt 3), 831–836. 10.1042/bj3090831 7639699 PMC1135707

[B94] Sheng J. Olrichs N. K. Geerts W. J. Li X. Rehman A. U. Gadella B. M. (2019). Zinc Binding Regulates Amyloid-like Aggregation of GAPR-1. Biosci. Rep. 39, BSR20182345. 10.1042/BSR20182345 30700571 PMC6900432

[B95] Sheng J. Gadella B. M. Olrichs N. K. Kaloyanova D. V. Helms J. B. (2021). The Less Conserved Metal-Binding Site in Human CRISP1 Remains Sensitive to Zinc Ions to Permit Protein Oligomerization. Sci. Rep. 11, 5498. 10.1038/s41598-021-84926-y 33750840 PMC7943821

[B96] Sun X. H. Zhu Y. Y. Wang L. Liu H. L. Ling Y. Li Z. L. (2017). The Catsper Channel and its Roles in Male Fertility: a Systematic Review. Reprod. Biol. Endocrinol. 15, 65. 10.1186/s12958-017-0281-2 28810916 PMC5558725

[B97] Tang W. Guo X. Niu L. Song D. Han B. Zhang H. (2020). Identification of Key Molecular Targets that Correlate with Breast Cancer through Bioinformatic Methods. J. Gene Med. 22, e3141. 10.1002/jgm.3141 31697007

[B98] Tapinos N. I. Polihronis M. Thyphronitis G. Moutsopoulos H. M. (2002). Characterization of the Cysteine-Rich Secretory Protein 3 Gene as an Early-Transcribed Gene with a Putative Role in the Pathophysiology of Sjögren's Syndrome. Arthritis Rheum. 46, 215–222. 10.1002/1529-0131(200201)46:1<215:AID-ART10024>3.0.CO;2-M 11817594

[B99] Turunen H. T. Sipilä P. Krutskikh A. Toivanen J. Mankonen H. Hämäläinen V. (2012). Loss of Cysteine-Rich Secretory Protein 4 (Crisp4) Leads to Deficiency in Sperm-Zona Pellucida Interaction in Mice. Biol. Reprod. 86, 1–8. 10.1095/biolreprod.111.092403 21865554

[B100] Udby L. Bjartell A. Malm J. Egesten A. Lundwall A. Cowland J. B. (2005). Characterization and Localization of Cysteine-Rich Secretory Protein 3 (CRISP-3) in the Human Male Reproductive Tract. J. Androl. 26, 333–342. 10.2164/jandrol.04132 15867000

[B101] Udby L. Cowland J. B. Johnsen A. H. Sørensen O. E. Borregaard N. Kjeldsen L. (2002). An ELISA for SGP28/CRISP-3, a Cysteine-Rich Secretory Protein in Human Neutrophils, Plasma, and Exocrine Secretions. J. Immunol. Methods 263, 43–55. 10.1016/s0022-1759(02)00033-9 12009203

[B102] Vadnais M. L. Foster D. N. Roberts K. P. (2008). Molecular Cloning and Expression of the CRISP Family of Proteins in the Boar. Biol. Reprod. 79, 1129–1134. 10.1095/biolreprod.108.070177 18716287

[B103] Volpert M. Furic L. Hu J. O'Connor A. E. Rebello R. J. Keerthikumar S. (2020). CRISP3 Expression Drives Prostate Cancer Invasion and Progression. Endocr. Relat. Cancer 27, 415–430. 10.1530/ERC-20-0092 32357309

[B104] Wang H. Yang H. Shivalila C. S. Dawlaty M. M. Cheng A. W. Zhang F. (2013). One-step Generation of Mice Carrying Mutations in Multiple Genes by CRISPR/Cas-mediated Genome Engineering. Cell 153, 910–918. 10.1016/j.cell.2013.04.025 23643243 PMC3969854

[B105] Weigel Muñoz M. Batisttone M. A. Carvajal G. Maldera J. A. Curci L. Torres P. (2018). Influence of the Genetic Background on the Reproductive Phenotype of Mice Lacking Cysteine-RIch Secretory Protein 1 (CRISP1). Biol. Rep. 99, 373–383. 10.1093/biolre/ioy048 29481619

[B106] Weigel Muñoz M. Carvajal G. Curci L. Gonzalez S. N. Cuasnicu P. S. (2019). Relevance of CRISP Proteins for Epididymal Physiology, Fertilization, and Fertility. Andrology 7, 610–617. 10.1111/andr.12638 31218833

[B107] Yamazaki Y. Brown R. L. Morita T. (2002). Purification and Cloning of Toxins from Elapid Venoms that Target Cyclic Nucleotide-Gated Ion Channels. Biochemistry 41, 11331–11337. 10.1021/bi026132h 12234174

[B108] Yamazaki Y. Morita T. (2004). Structure and Function of Snake Venom Cysteine-Rich Secretory Proteins. Toxicon 44, 227–231. 10.1016/j.toxicon.2004.05.023 15302528

[B109] Yanagimachi R. (1994). “Mammalian Fertilization,”. The Physiology Of Reproduction. Editors Knobil E. Neill. J. D., 189–317.

[B110] Zhang C. Zhou Y. Xie S. Yin Q. Tang C. Ni Z. (2018). CRISPR/Cas9-mediated Genome Editing Reveals the Synergistic Effects of β-defensin Family Members on Sperm Maturation in Rat Epididymis. FASEB J. 32, 1354–1363. 10.1096/fj.201700936R 29141997

[B111] Zhang M. Bromfield E. G. Veenendaal T. Klumperman J. Helms J. B. Gadella B. M. (2021). Characterization of Different Oligomeric Forms of CRISP2 in the Perinuclear Theca versus the Fibrous Tail Structures of Boar Spermatozoa. Biol. Reprod. 26, ioab145. 10.1093/biolre/ioab145 34309660

[B112] Zhou J. H. Zhou Q. Z. Lyu X. M. Zhu T. Chen Z. J. Chen M. K. (2015). The Expression of Cysteine-Rich Secretory Protein 2 (CRISP2) and its Specific Regulator miR-27b in the Spermatozoa of Patients with Asthenozoospermia. Biol. Reprod. 92, 28–29. 10.1095/biolreprod.114.124487 25505194

[B113] Zhou Y. S. Webb S. Lettice L. Tardif S. Kilanowski F. Tyrrell C. (2013). Partial Deletion of Chromosome 8 β-defensin Cluster Confers Sperm Dysfunction and Infertility in Male Mice. Plos Genet. 9, e1003826. 10.1371/journal.pgen.1003826 24204287 PMC3812073

